# Letter to the Editor: Cancer rates not explained by smoking: how to investigate a single county

**DOI:** 10.1186/s12940-021-00737-8

**Published:** 2021-05-21

**Authors:** Douglas J. Myers, Polly Hoppin, Molly Jacobs, Richard Clapp, David Kriebel

**Affiliations:** 1 Department of Community and Environmental Health, College of Health Sciences, Boise State University, 1910 University Drive, Idaho 83725 Boise, USA; 2 Lowell Center for Sustainable Production, University of Massachusetts, Lowell 1 University Avenue, 01854 Lowell, MA USA

To the Editor:

We recently published Cancer rates not explained by smoking: a county-level analysis in your journal [1]. Using U.S. Surveillance, Epidemiology and End Results (SEER) cancer incidence and population data for 612 counties [2], we simulated the expected effect of eliminating smoking on the rates of 12 types of cancer known to be caused by smoking [3]. We estimated that in 2016, 39.8% of the cancer incidence of these 12 types would not have occurred had smoking been eliminated. Conversely, about 60% of these cancers would still occur, even in the absence of smoking. This finding is in good agreement with previously published estimates of the attributable fraction of cancer due to smoking [4].

A novel finding from our county-level approach was that there would be considerable variability in the benefit of smoking elimination; and in particular that some counties would benefit only modestly from the best case scenario of tobacco control. We found that under this scenario, SEER counties whose cancer incidence of the 12 smoking-related types would decline by less than 10% were in the metropolitan areas of large cities such as Detroit MI, Louisville KY and New Orleans LA.

Following a symposium on cancer and environment in Pittsburgh PA in 2019^1^, we were asked by public health and community leaders to apply this analysis to their county, Allegheny. The Pittsburgh region has long suffered from elevated levels of air pollution, and members of the public are concerned about the risk it poses to public health.

Allegheny County is not in SEER, so additional analyses were needed to answer this question. Here we describe briefly how we applied our analysis of hypothetical smoking elimination to a specific non-SEER county. In the online supplement, we provide a step-by-step summary of the method and the key computer code, in STATA [5], to allow others to investigate their county, whether or not it is in SEER. We demonstrate using the method with lung cancer alone rather than with the set of 12 smoking-related types that were the focus of our paper; in practice, any set of cancers could be used.

We obtained the counts of lung cancers in Allegheny County by age and gender from the PA Cancer Registry 2, and population data from the US Census Bureau for 2016. Smoking data were available from Institute for Health Metrics and Evaluation (IHME) [6]. We chose to lag smoking by 20 years, the most commonly applied lag for smoking and lung cancer, although other lags could have been chosen. We fit the same multi-level mixed-effects regression model described in our paper [1] to the augmented dataset of 613 counties (SEER+Allegheny). This allowed us to estimate the 2016 incidence rate of lung cancer under the scenario of complete smoking elimination for the last 20 years.

As expected, smoking elimination would have had a substantial effect on lung cancer incidence across the SEER counties in 2016, with an average reduction of 62%, consistent with tobaccos well-known status as the predominant cause of lung cancer. However, the model predicts a substantial variation in this reduction from county to county; the top 5% of counties would experience a reduction greater than 80%, while the bottom 5% would see a reduction of less than 15% in lung cancer incidence from smoking elimination. Allegheny County is in this latter category: the model estimated that Allegheny Countys lung cancer rate would have dropped by only 11%, meaning less benefit in terms of reduced lung cancer rates than in all but 2% of SEER counties.

Communities often want to know why is our cancer rate elevated? Epidemiology is frequently unable to provide clear answers to this question because of small numbers, difficulties characterizing historical exposures for long latency diseases, and other well-known pitfalls of cluster investigations [7]. The analyses described here do not answer this basic question, but may help move past a frequent sticking point in local discussions. A common response from authorities is that smoking is the single most important cause of cancer, particularly for lung cancer, with the implication that environmental risk factors should not be priority concerns. This is despite known environmental and occupational causes of lung cancer, including urban air pollution [8] and dozens of industrial chemicals in occupational settings [9].

The ranking of Allegheny County in the bottom 2% of SEER counties with regard to the benefit of tobacco control for lung cancer raises a red flag about past and ongoing exposures that may increase cancer risk. Indeed, in Allegheny County, radon is of concern, as are pollutants known to cause lung and other types of cancer which are emitted from manufacturing (coke ovens are a particular concern in Pittsburgh) and mobile sources, at levels that put residents at higher risk than in most other counties in the country [10].

Our recently-published paper established a method for identifying counties where public health authorities should be particularly attuned to risk factors for cancer other than tobacco, in addition to their ongoing efforts to reduce smoking [1]. The attached Supplement describes methods for identifying these counties. As noted in the original manuscript, a limitation of this approach is the lack of individual-level smoking data (we refer readers to the manuscript for additional discussion of limitations) [1]. In the context of environmental justice, this approach may assist public health, environmental and community leaders in addressing disparate burdens of exposures among counties. Where counties or communities are still exposed to carcinogens in air, water and workplaces, we encourage aggressive investment in reducing exposure and promoting safer alternatives as essential pathways for cancer prevention.

## Supplementary information

Additional file 1 Supplementary material

## Data Availability

Dataset analyzed during the current study is publicly available at the Surveillance, Epidemiology and End Results Program (SEER) repository, https://seer.cancer.gov/data/.

## Abbreviations

SEER: Surveillance, Epidemiology and End Results

## Footnotes

1 https://sites.google.com/silentspring.org/cancer-environment-pasymposium/home

2 These data were provided by the Pennsylvania Department of Health. The Department specifically disclaims responsibility for any analyses, interpretations or conclusions.
