# Supplementary material for: Letter to the Editor: Cancer rates not explained by smoking: how to investigate a single county
Source: Environ Health. 2021 May 21;20:62. doi: 10.1186/s12940-021-00737-8 (PMC8139076; doi:10.1186/s12940-021-00737-8)
Supplement: Supplementary file 1 — Supplementary material [file 12940_2021_737_MOESM1_ESM.docx]

**Supplementary material to**

**Letter to the Editor: Cancer rates not explained by smoking: how to investigate a single county.**

Douglas J. Myers^1^*, Polly Hoppin^2^, Molly Jacobs^2^, Richard Clapp^2^ and David Kriebel^2^

**Summary**

Researchers can use statistical software to estimate the impact of eliminating an exposure, such as smoking, on one or more cancer types. Models can be used to simulate hypothetical results for any chosen exposure level and compare them to results using actual exposure levels. In the research we presented in [1], we used STATA Statistics/Data Software Program, version 16.1. The procedure outlined below illustrates the method using county-level smoking prevalence as the exposure. It is assumed that researchers have acquired and prepared the SEER incidence and population data.

**Data Needs**

Incidence and Population Data

Cancer incidence data (counts) grouped by year, county, sex and age groups (our model used five-year age groups from 20 to 85), and related population data, both available from the SEER program [2], form the basis of the modeling which is used to generate actual and simulated predicted results. An external population distribution corresponding to the age-group and sex cells utilized, and the population total, are needed to perform direct standardization. We chose all 2016 SEER counties combined as our population distribution used in the direct standardization procedure.

If non-SEER counties are to be investigated, cancer incidence data stratified by the same sex and age groups are obtained from the appropriate cancer registry, and these are merged with the data for the SEER counties.

Exposure Data

We obtained county-level sex-stratified smoking prevalence data for all US counties, from 1996 to 2012, from the Institute for Health Metrics and Evaluation [6]. To generate models and obtain predicted results, exposure data, appropriately lagged, should be merged by county, sex (if sex specific) and year with the incidence and population data, both SEER and, if necessary, one’s own non-SEER county units.

**Regression Models**

Once the incidence, population and exposure data are prepared, regression techniques can be used to model the effects of exposure in addition to common confounding variables (e.g., age and sex) and other variables of interest (e.g., year). We used a multilevel negative binomial regression model to model our data. Researchers will need to determine the appropriate model for their outcome (negative binomial, Poisson, etc.) and data structure (ordinary or hierarchical models).

Our outcome was counts of smoking-related cancers by cell (based on age, sex, county and year). We modeled year and age groups as sets of indicator variables, sex as a dichotomous variable, and county-level smoking prevalence (lagged) as a continuous variable. The relevant STATA code is:

menbreg cancer_incidence i.year i.age_group sex county_smoking_prevalence_lagged, exp(cell_population) || county: R.year, irr

Cell size population was used as the offset (**exp(cell_population)**). Random intercepts were allowed for each county (**|| county:**) and random slopes were allowed for year (**R.year**). Finally, **irr** yielded incidence rate ratios instead of coefficients. [Note that all STATA commands are single lines of code.]

**Predicted Values**

The next step is to get the predicted count of the cancers by cell. In STATA, this is done with the **predict** command. For negative binomial regression models, the cell count is the default value created by this command. The results were stored in a variable called *counts_yhat*:

predict counts_yhat

Then, the exposure data variable (*county_smoking_prevalence_lagged*) was replaced with zero, or whatever value the researcher wishes to assign to all counties, using STATA’s **replace** command:

replace county_smoking_prevalence_lagged = 0

The **predict** command was run again. This time, the predicted values were generated by applying the model parameters previously created to the same variables as were included in the model, but using the new values assigned to the exposure variable:

predict counts_yhat0

**Direct Standardization**

These predicted counts were then directly standardized to remove the effect of varying population age distributions across county and year. Predicted counts were turned into rates by dividing them by the cell population. These rates were multiplied by the external referent population distribution pertaining to each corresponding cell based on age group and gender. These two steps were done in one command. STATA’s **generate** command was used to create two new variables – the standardized incidence rates for the results with smoking models using actual values (*incidence_yhat_rate_std*) and with values equal to zero for all cells (*incidence_yhat0_rate_std*):

generate incidence_yhat_rate_std = (counts_yhat/cell_population)*seer_cell_population_2016

generate incidence_yhat0_rate_std = (counts_yhat0/cell_population)*seer_cell_population_2016

We used the 2016 SEER counties’ population as the external weight age distribution. Final rates produced will be sensitive to the choice of age distribution based on geography and/or time. However, county rates produced by this procedure using a common external weight distribution can be compared to each other and across time.

Both of these weighted predicted rates, along with the external population weights, were then summed by year and county, before the final step in standardization was performed. This produced one value for each county per year. We used STATA’s **collapse** command:

collapse (sum) incidence_yhat_rate_std incidence_yhat0_rate_std seer_population_16, by(year county)

Then these county totals (for each year) were divided by the external population totals (the final step in direct standardization) and multiplied by 100,000 to get the county-level predicted rates per 100,000 person-years:

generate incidence_yhat_rate_std _final = incidence_yhat_rate_std/seer_population_16*100000

generate incidence_yhat0_rate_std _final = incidence_yhat0_rate_std/seer_population_16*100000

The predicted values, both actual and simulated, can now be compared by county. STATA’s **list** command can be used to display the county identifier and the two standardized predicted rates for 2016.

list county incidence_yhat_rate_std _final incidence_yhat0_rate_std _final if year==2016
